# Supplementary figures and images for: Proteome-scale relationships between local amino acid composition and protein fates and functions
Source: PLoS Comput Biol. 2018 Sep 24;14(9):e1006256. doi: 10.1371/journal.pcbi.1006256 (PMC6171957; doi:10.1371/journal.pcbi.1006256)

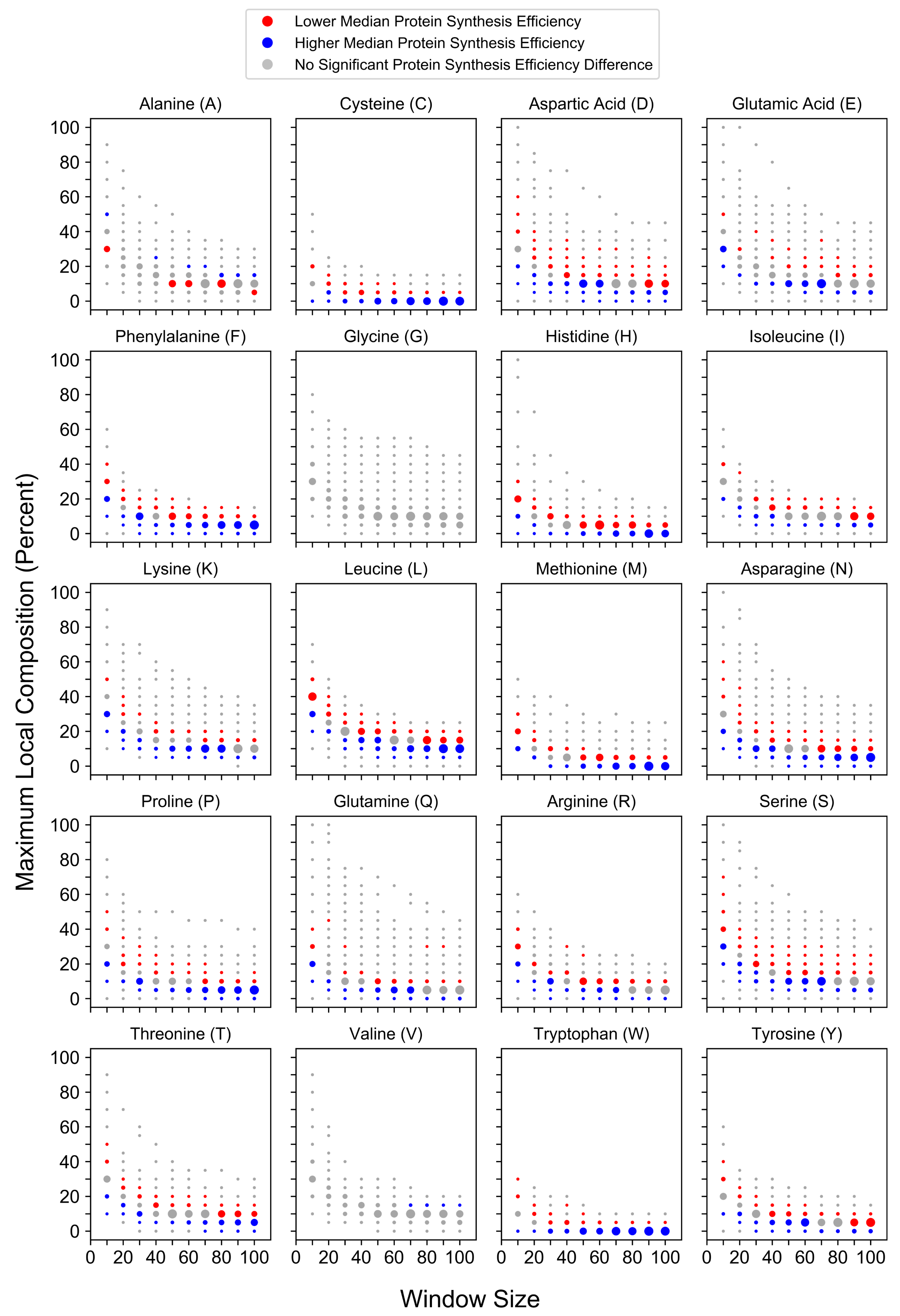

Supplement: S1 Fig — Local enrichment for individual amino acids correspond to composition-dependent changes in experimentally-derived protein synthesis efficiency [56]. (TIF) [file pcbi.1006256.s004.tif]

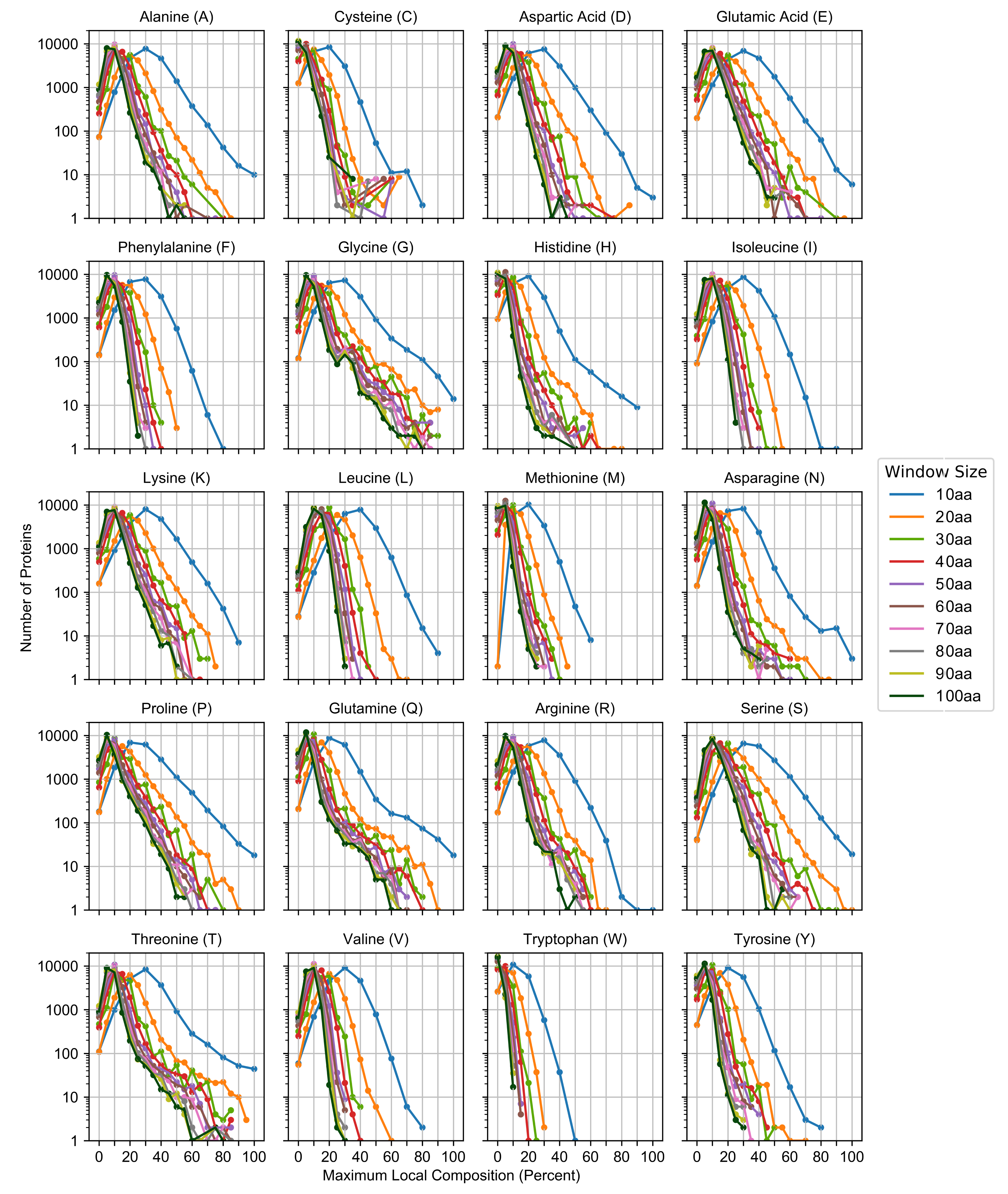

Supplement: S2 Fig — The number of proteins partitioned into each window size/percent composition bin for each of the 20 canonical amino acids are plotted as a function of maximum local composition for each window size. Scatter points are connected by line segments for visual clarity only. (TIF) [file pcbi.1006256.s005.tif]

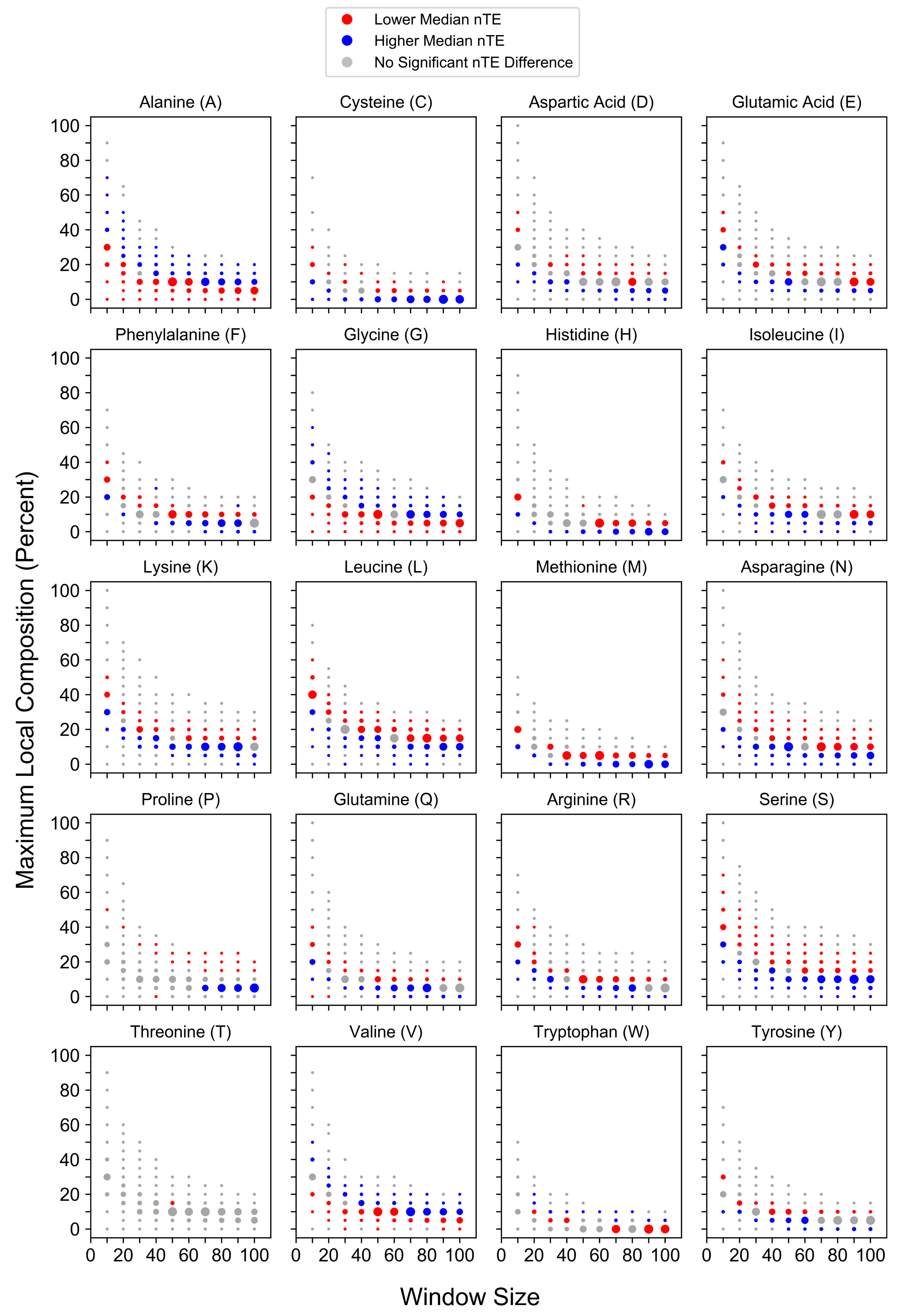

Supplement: S3 Fig — For each amino acid, nTE values corresponding to proteins partitioned into a given window size and percent composition bin were compared to values for all proteins of length ≥ the corresponding window size that were excluded from the bin. Red and blue points indicate bins for which the distribution of protein half-life values differ significantly (Bonferroni-corrected p ≤ 0.05) from those of excluded proteins: red points indicate bins with a lower median value relative to that of excluded proteins, whereas blue points indicated bins with a higher relative median value. Grey points indicate comparisons lacking statistical significance. Individual points are scaled within each subplot to reflect the sample sizes of proteins contained within each bin. (TIF) [file pcbi.1006256.s006.tif]

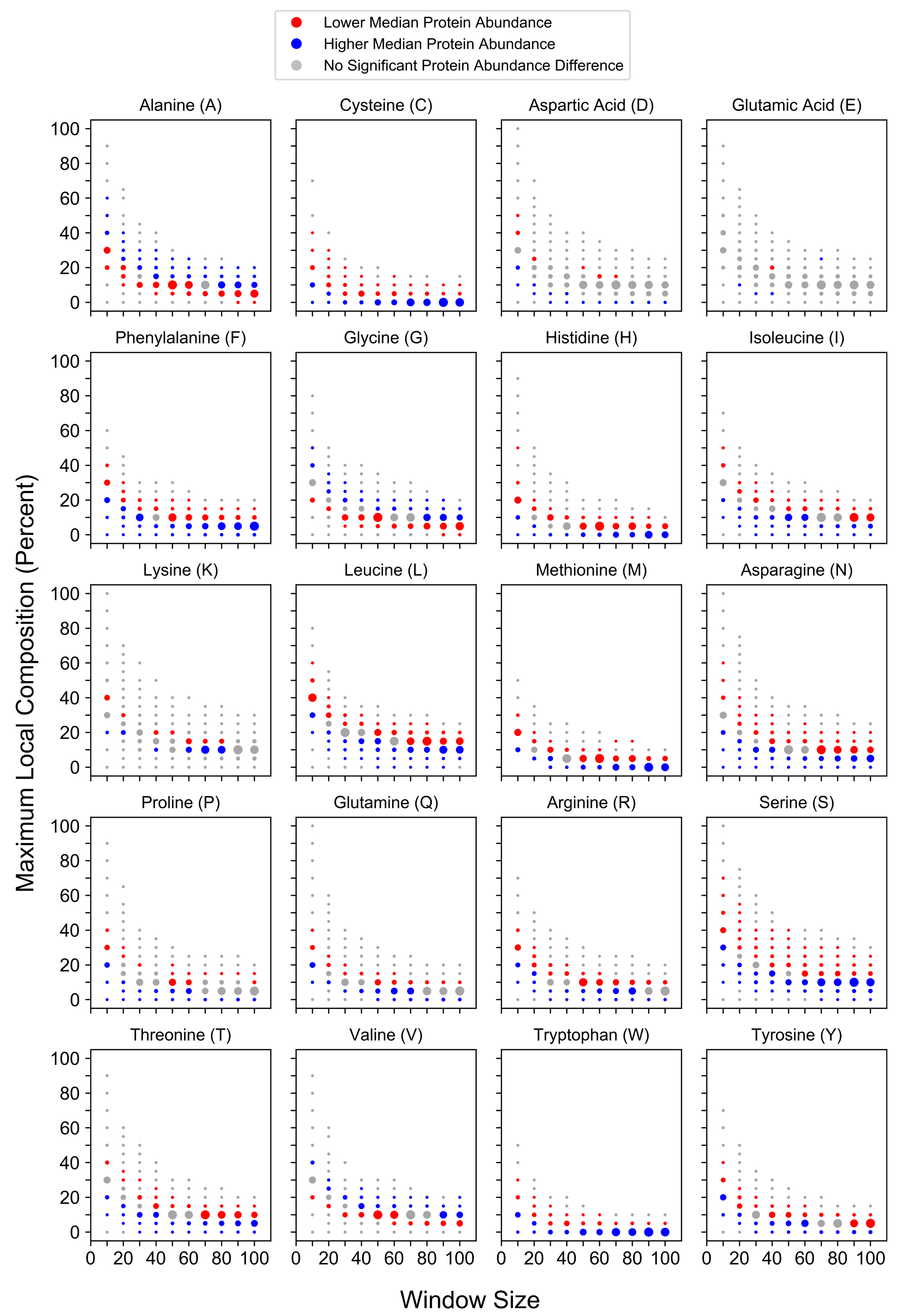

Supplement: S4 Fig — For each amino acid, protein abundance values corresponding to proteins partitioned into a given window size and percent composition bin were compared to values for all proteins of length ≥ the corresponding window size that were excluded from the bin. Red and blue points indicate bins for which the distribution of protein half-life values differ significantly (Bonferroni-corrected p ≤ 0.05) from those of excluded proteins: red points indicate bins with a lower median value relative to that of excluded proteins, whereas blue points indicated bins with a higher relative median value. Grey points indicate comparisons lacking statistical significance. Individual points are scaled within each subplot to reflect the sample sizes of proteins contained within each bin. (TIF) [file pcbi.1006256.s007.tif]

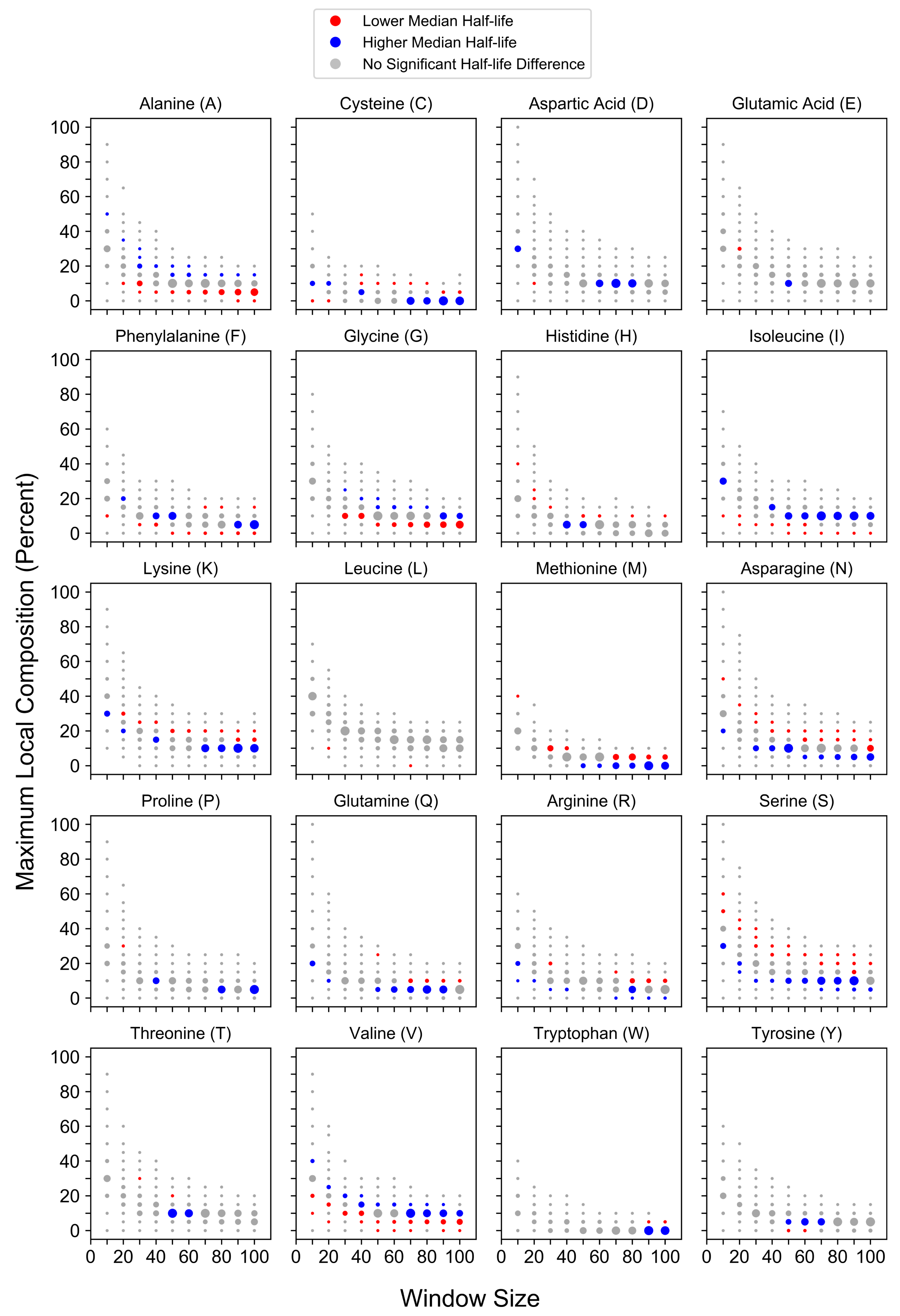

Supplement: S5 Fig — For each amino acid, protein half-life values corresponding to proteins partitioned into a given window size and percent composition bin were compared to values for all proteins of length ≥ the corresponding window size that were excluded from the bin. Red and blue points indicate bins for which the distribution of protein half-life values differ significantly (Bonferroni-corrected p ≤ 0.05) from those of excluded proteins: red points indicate bins with a lower median value relative to that of excluded proteins, whereas blue points indicated bins with a higher relative median value. Grey points indicate comparisons lacking statistical significance. Individual points are scaled within each subplot to reflect the sample sizes of proteins contained within each bin. (TIF) [file pcbi.1006256.s008.tif]

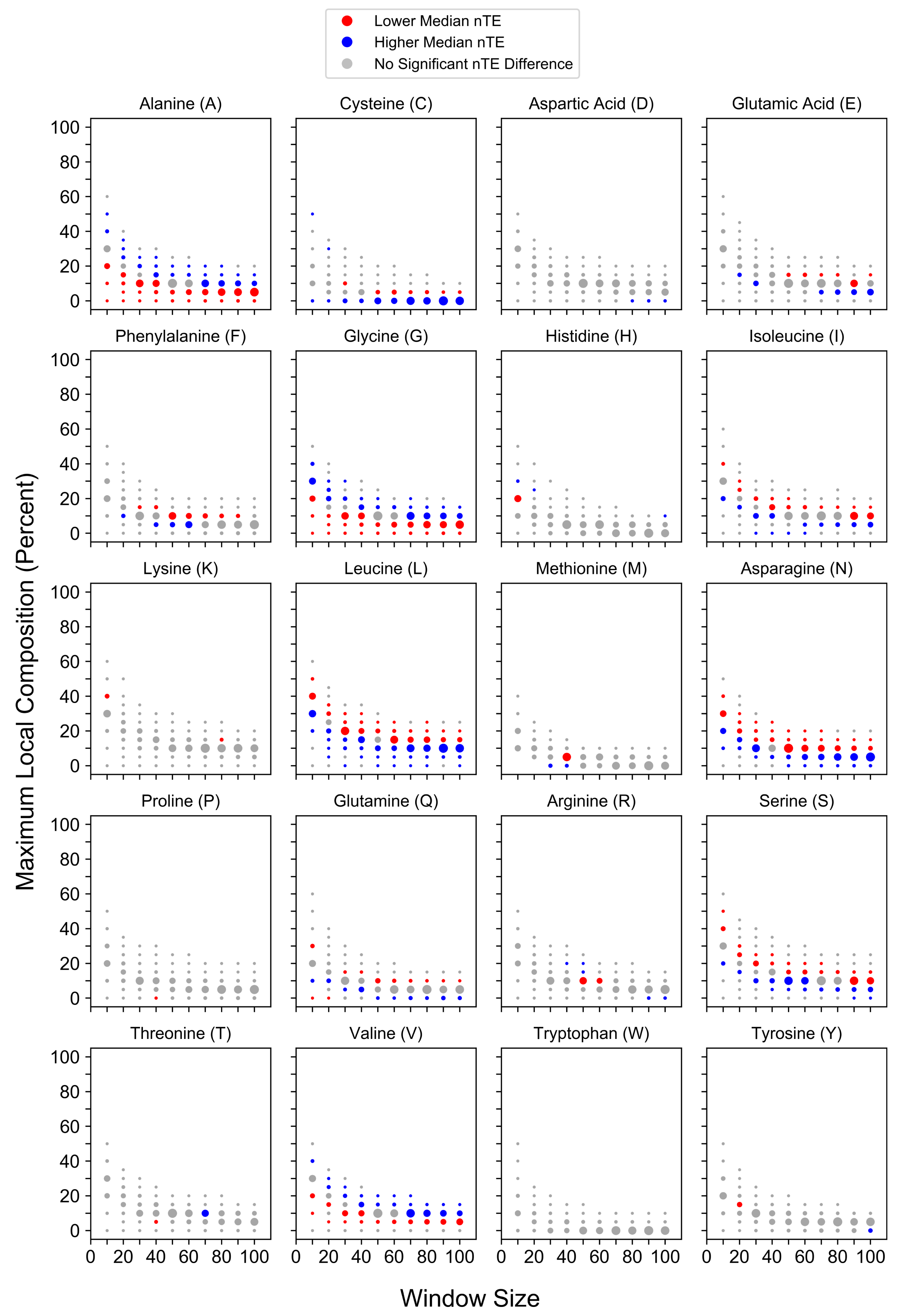

Supplement: S6 Fig — For each amino acid, nTE values corresponding to proteins partitioned into a given window size and percent composition bin were compared to values for all proteins of length ≥ the corresponding window size that were excluded from the bin. Red and blue points indicate bins for which the distribution of protein half-life values differ significantly (Bonferroni-corrected p ≤ 0.05) from those of excluded proteins: red points indicate bins with a lower median value relative to that of excluded proteins, whereas blue points indicated bins with a higher relative median value. Grey points indicate comparisons lacking statistical significance. Individual points are scaled within each subplot to reflect the sample sizes of proteins contained within each bin. (TIF) [file pcbi.1006256.s009.tif]

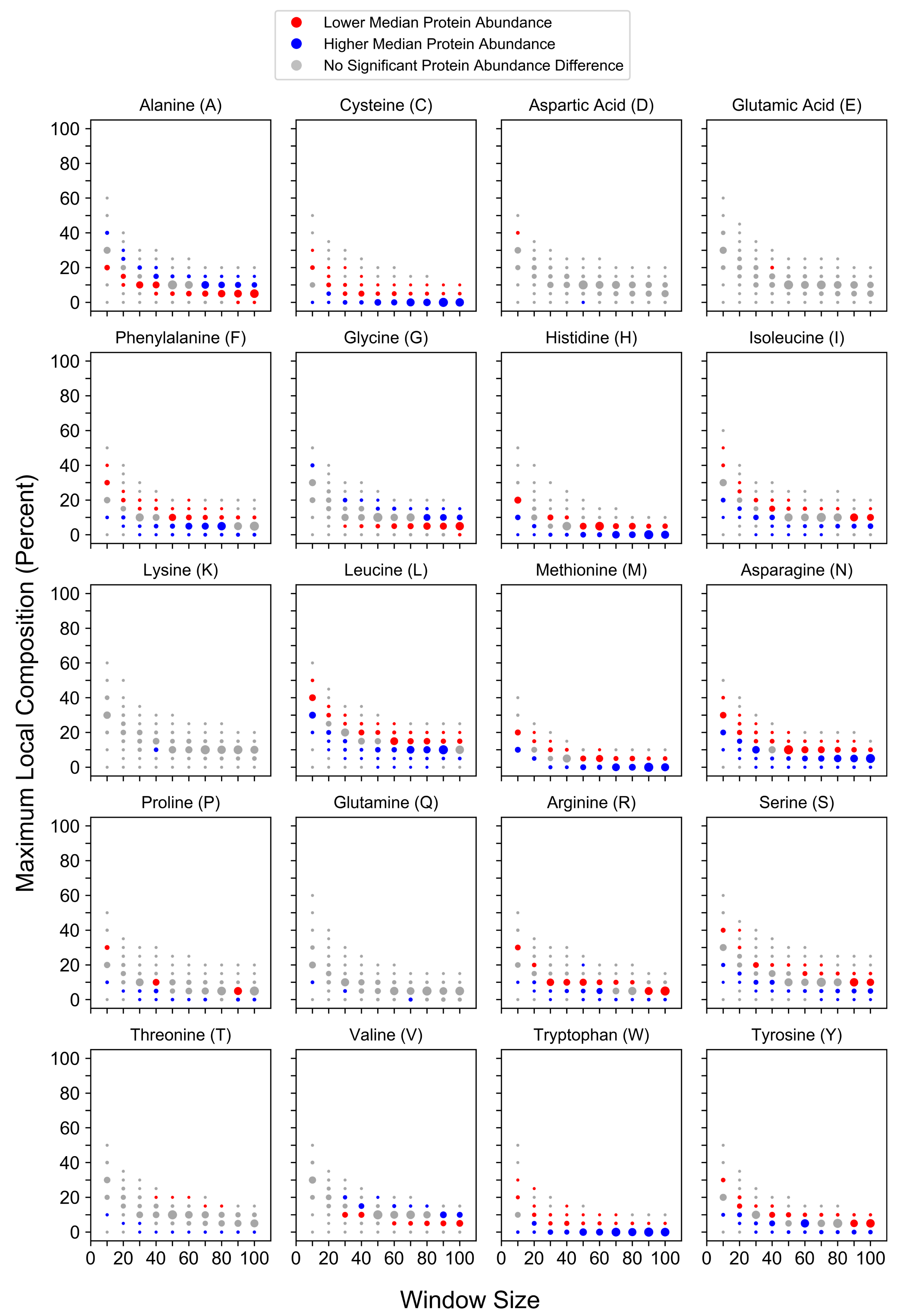

Supplement: S7 Fig — For each amino acid, protein abundance values corresponding to proteins partitioned into a given window size and percent composition bin were compared to values for all proteins of length ≥ the corresponding window size that were excluded from the bin. Red and blue points indicate bins for which the distribution of protein half-life values differ significantly (Bonferroni-corrected p ≤ 0.05) from those of excluded proteins: red points indicate bins with a lower median value relative to that of excluded proteins, whereas blue points indicated bins with a higher relative median value. Grey points indicate comparisons lacking statistical significance. Individual points are scaled within each subplot to reflect the sample sizes of proteins contained within each bin. (TIF) [file pcbi.1006256.s010.tif]

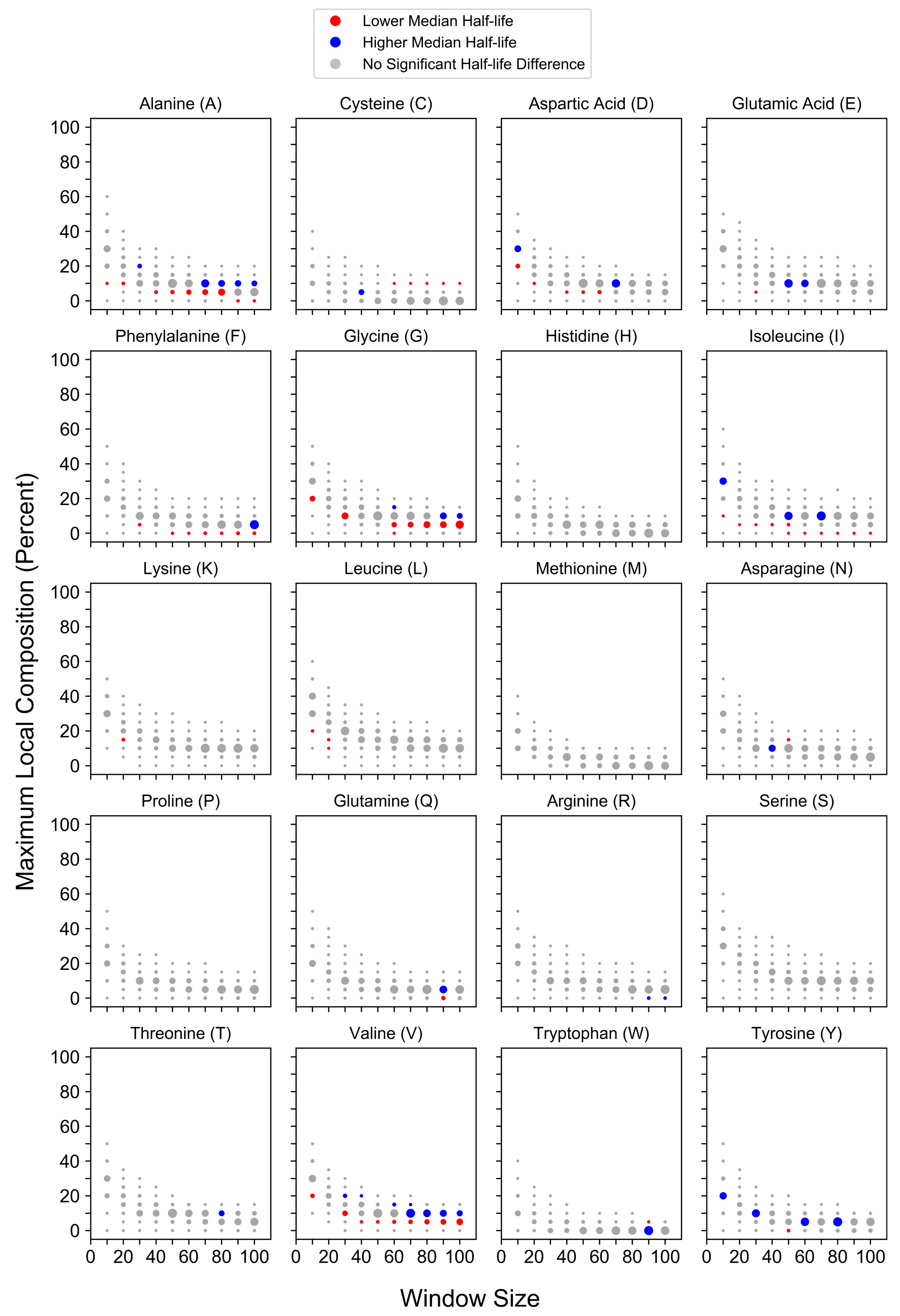

Supplement: S8 Fig — For each amino acid, protein half-life values corresponding to proteins partitioned into a given window size and percent composition bin were compared to values for all proteins of length ≥ the corresponding window size that were excluded from the bin. Red and blue points indicate bins for which the distribution of protein half-life values differ significantly (Bonferroni-corrected p ≤ 0.05) from those of excluded proteins: red points indicate bins with a lower median value relative to that of excluded proteins, whereas blue points indicated bins with a higher relative median value. Grey points indicate comparisons lacking statistical significance. Individual points are scaled within each subplot to reflect the sample sizes of proteins contained within each bin. (TIF) [file pcbi.1006256.s011.tif]

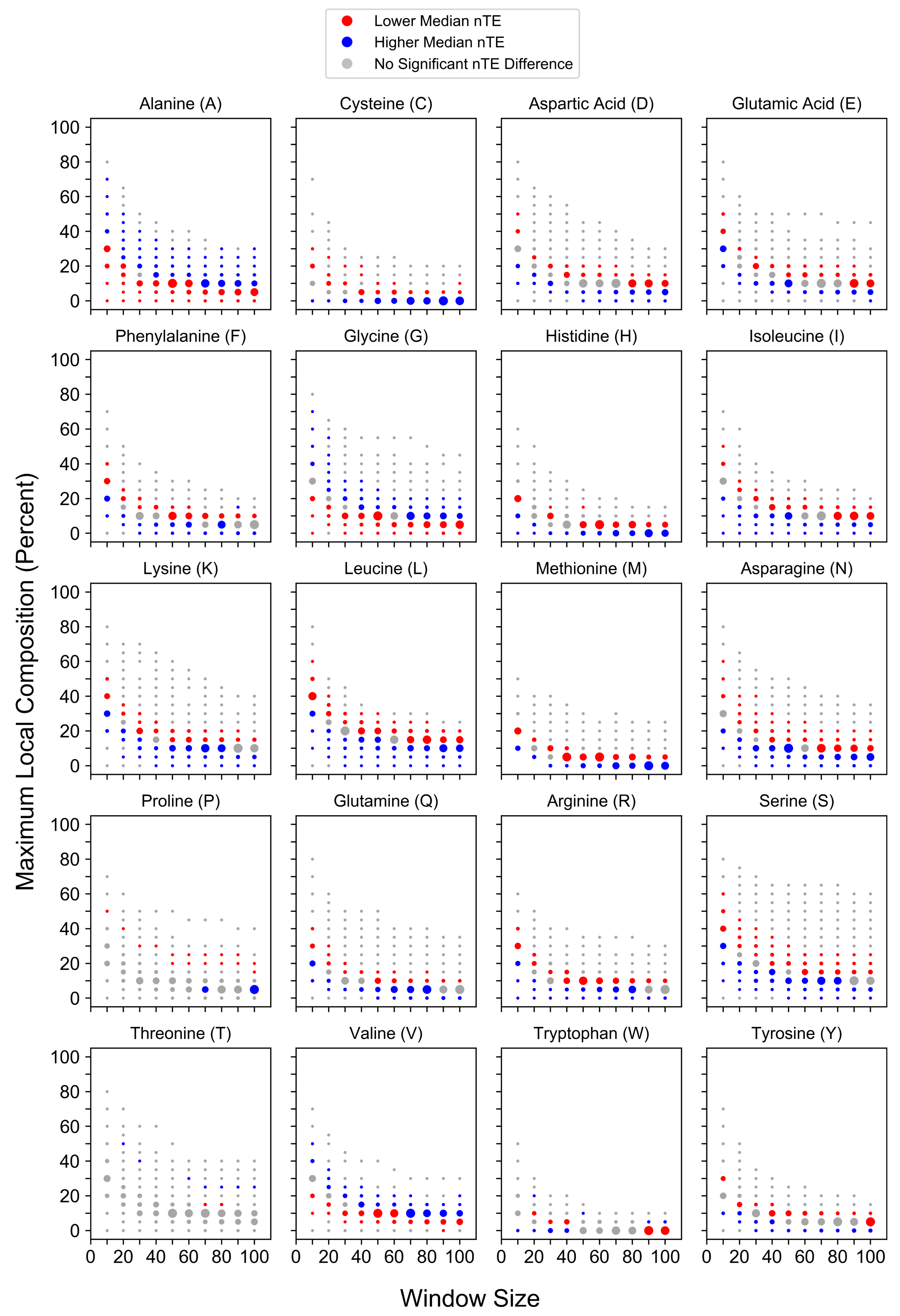

Supplement: S9 Fig — For each amino acid, nTE values corresponding to proteins partitioned into a given window size and percent composition bin were compared to values for all proteins of length ≥ the corresponding window size that were excluded from the bin. Red and blue points indicate bins for which the distribution of protein half-life values differ significantly (Bonferroni-corrected p ≤ 0.05) from those of excluded proteins: red points indicate bins with a lower median value relative to that of excluded proteins, whereas blue points indicated bins with a higher relative median value. Grey points indicate comparisons lacking statistical significance. Individual points are scaled within each subplot to reflect the sample sizes of proteins contained within each bin. (TIF) [file pcbi.1006256.s012.tif]

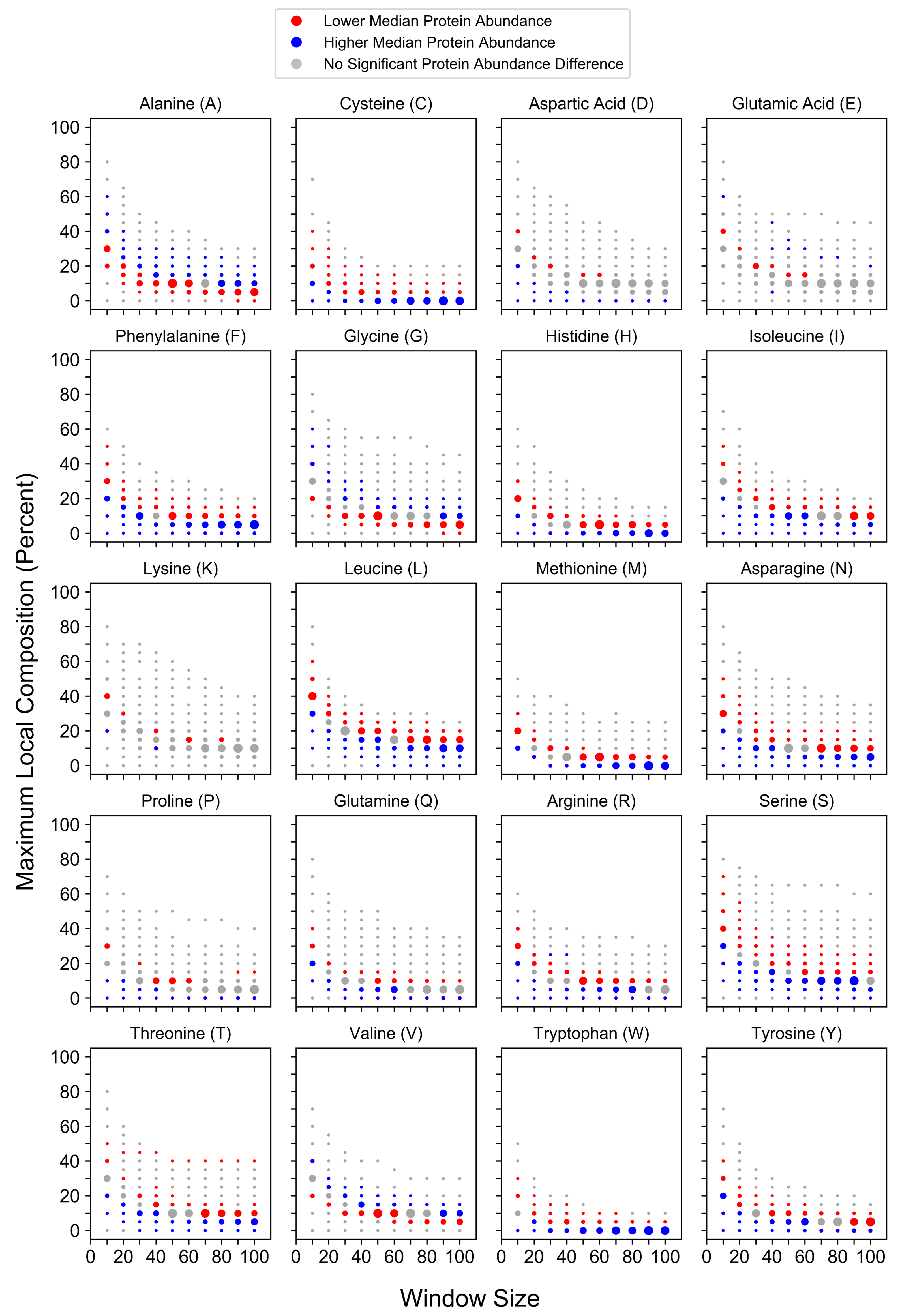

Supplement: S10 Fig — For each amino acid, protein abundance values corresponding to proteins partitioned into a given window size and percent composition bin were compared to values for all proteins of length ≥ the corresponding window size that were excluded from the bin. Red and blue points indicate bins for which the distribution of protein half-life values differ significantly (Bonferroni-corrected p ≤ 0.05) from those of excluded proteins: red points indicate bins with a lower median value relative to that of excluded proteins, whereas blue points indicated bins with a higher relative median value. Grey points indicate comparisons lacking statistical significance. Individual points are scaled within each subplot to reflect the sample sizes of proteins contained within each bin. (TIF) [file pcbi.1006256.s013.tif]

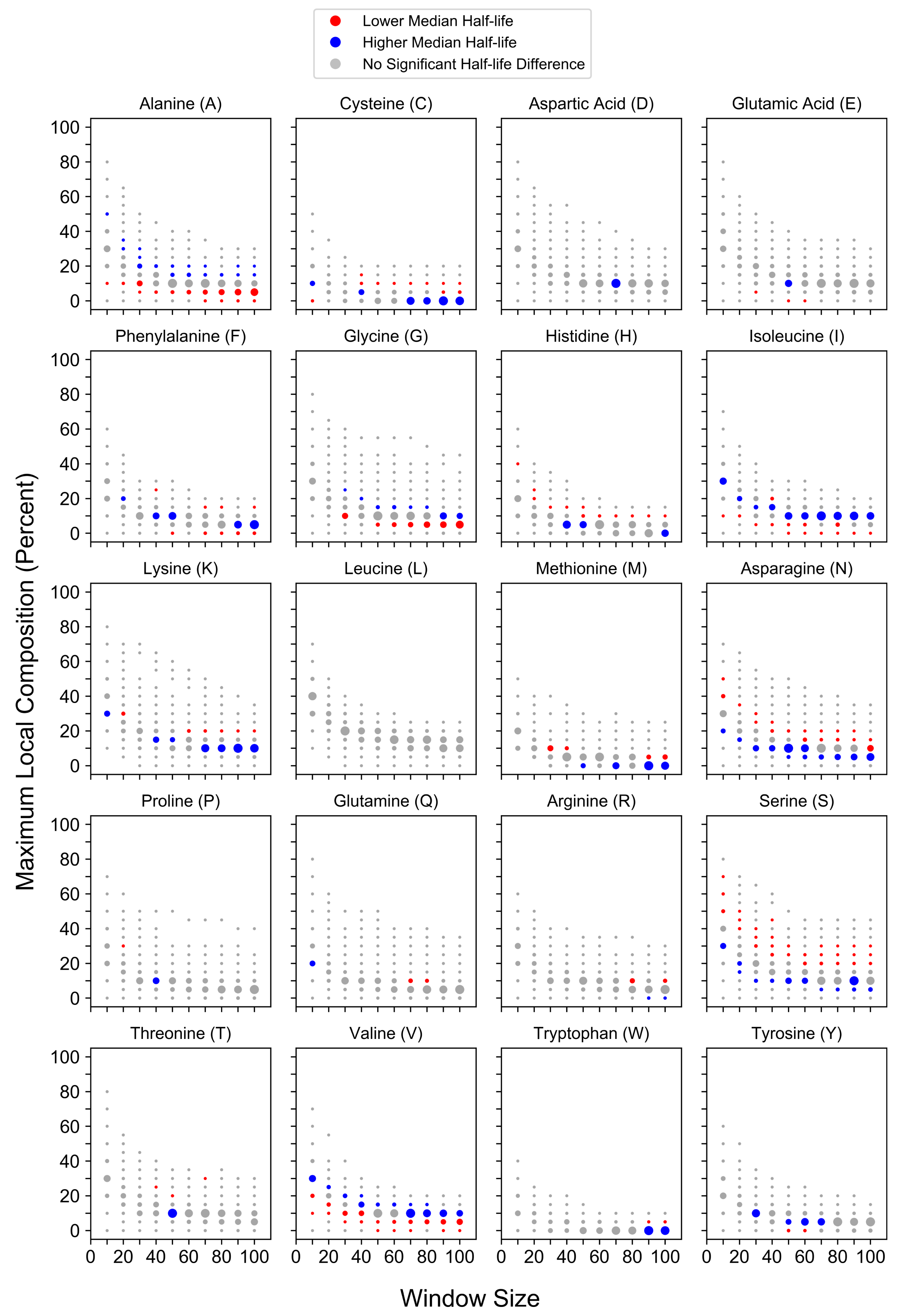

Supplement: S11 Fig — For each amino acid, protein half-life values corresponding to proteins partitioned into a given window size and percent composition bin were compared to values for all proteins of length ≥ the corresponding window size that were excluded from the bin. Red and blue points indicate bins for which the distribution of protein half-life values differ significantly (Bonferroni-corrected p ≤ 0.05) from those of excluded proteins: red points indicate bins with a lower median value relative to that of excluded proteins, whereas blue points indicated bins with a higher relative median value. Grey points indicate comparisons lacking statistical significance. Individual points are scaled within each subplot to reflect the sample sizes of proteins contained within each bin. (TIF) [file pcbi.1006256.s014.tif]

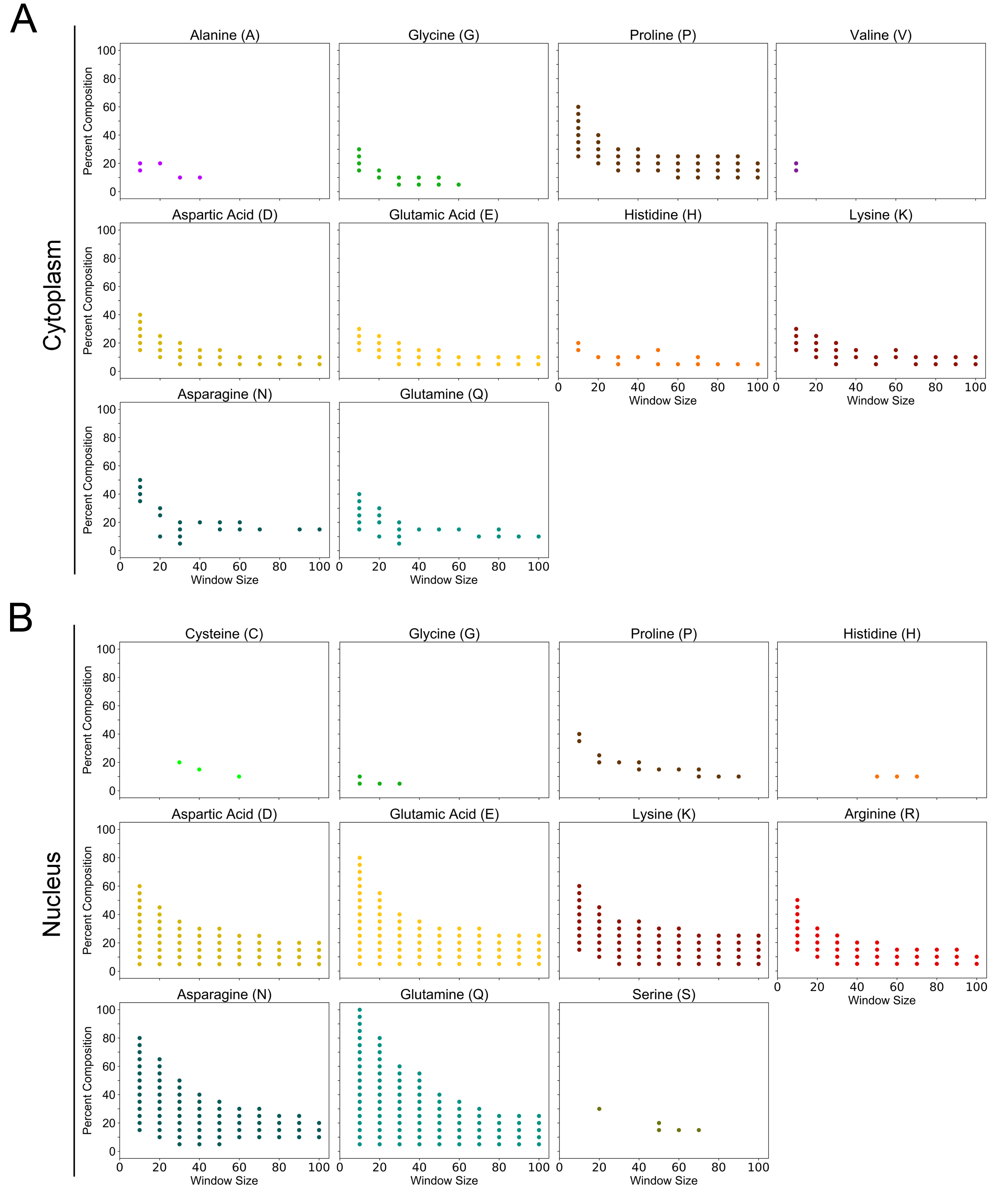

Supplement: S12 Fig — Composition ranges for each amino acid significantly associated with the cytoplasm (A) and nucleus (B) are indicated. All plotted points indicate protein sets for which association with the indicated subcellular compartment is statistically significant (Bonferonni-corrected p < 0.05). Plots are shown only for amino acids with at least two composition bins significantly associated with the indicated subcellular compartment. (TIF) [file pcbi.1006256.s015.tif]

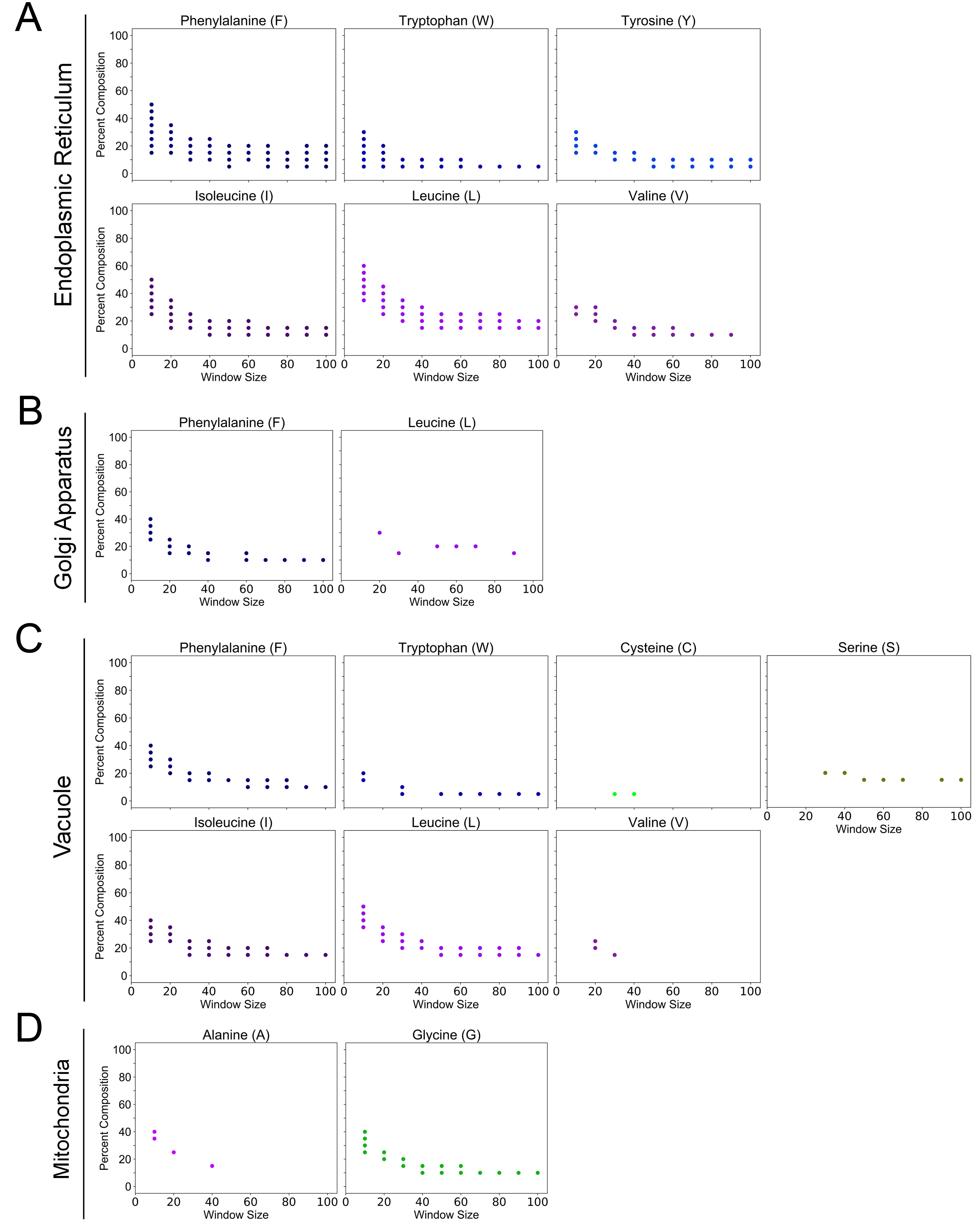

Supplement: S13 Fig — Composition ranges for each amino acid significantly associated with the endoplasmic reticulum (A), Golgi apparatus (B), vacuole (C), and mitochondria (D) are indicated. All plotted points indicate protein sets for which association with the indicated subcellular compartment is statistically significant (Bonferonni-corrected p < 0.05). Plots are shown only for amino acids with at least two composition bins significantly associated with the indicated subcellular compartment. (TIF) [file pcbi.1006256.s016.tif]

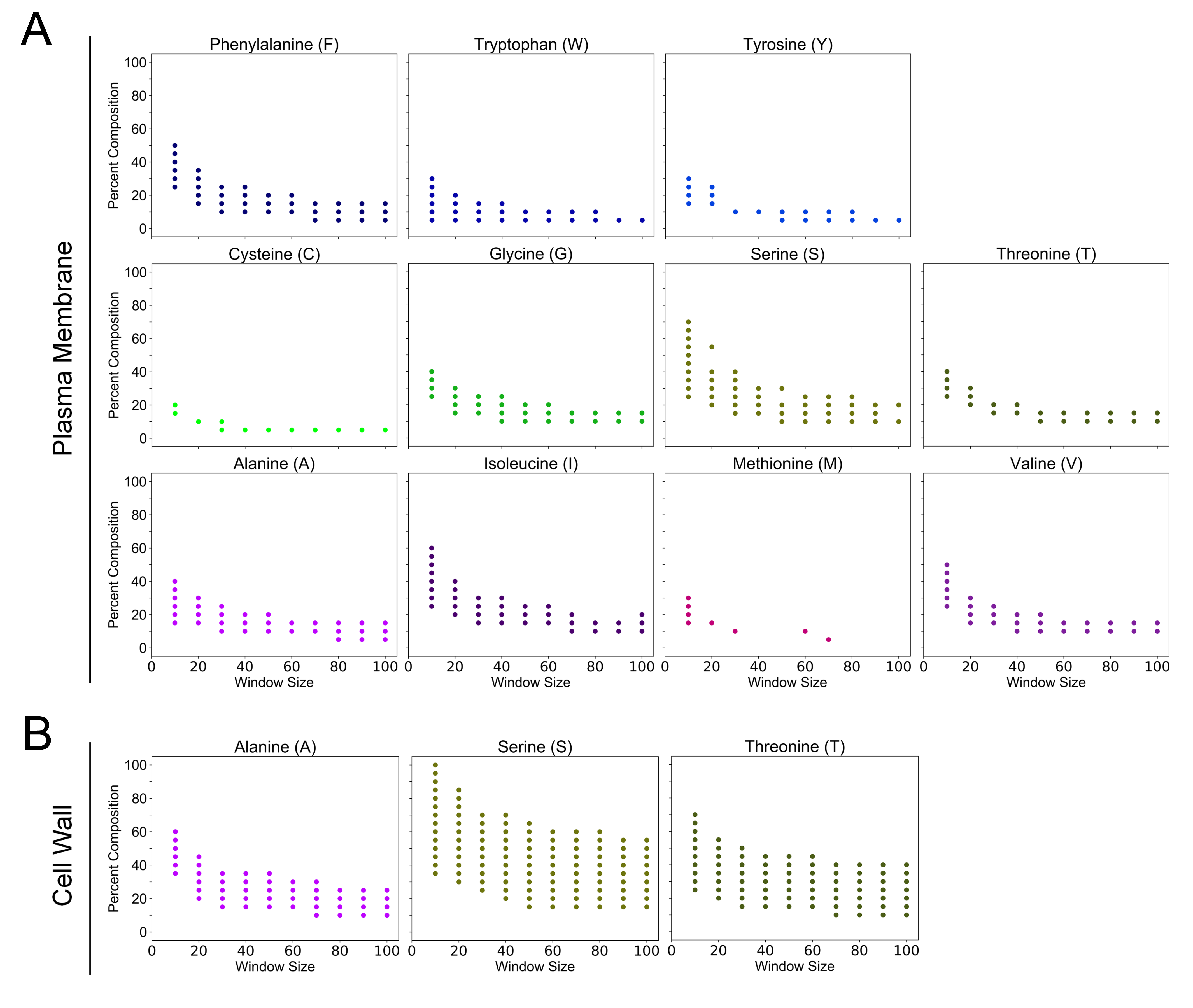

Supplement: S14 Fig — Composition ranges for each amino acid significantly associated with the plasma membrane (A) and cell wall (B) are indicated. All plotted points indicate protein sets for which association with the indicated subcellular compartment is statistically significant (Bonferonni-corrected p < 0.05). Plots are shown only for amino acids with at least two composition bins significantly associated with the indicated subcellular compartment. (TIF) [file pcbi.1006256.s017.tif]
